# Complete mitochondrial genome of *Salix variegata* Franch.: assembly, characterization, and comparative analysis

**DOI:** 10.3389/fpls.2026.1804453

**Published:** 2026-06-19

**Authors:** Huan Zhang, Rui Pan, Jingyu Zeng, Wenqiao Li, Xiao Zhang, Mingwei Tang, Hongping Deng

**Affiliations:** 1 School of Life Sciences, Southwest University, Beibei District, Chongqing, China; 2 Department of Pharmacy and Biotechnology (FABIT), University of Bologna, Bologna, Italy

**Keywords:** codon preference, phylogeny, plant mitogenome, repeat sequences, Salicaceae

## Abstract

**Introduction:**

*Salix variegata* is a widely distributed species in the genus *Salix* with high ecological restoration and ornamental value. Nevertheless, only the chloroplast genome of this species has been published, and its mitochondrial genome has not yet been explored.

**Methods:**

In this study, we assembled and annotated the complete mitochondrial genome of *S. variegata* for the first time. A comprehensive analysis was further conducted on its structural characteristics, repetitive sequences, predicted RNA editing sites, codon usage preferences, intracellular gene transfer, and phylogenetic inference.

**Results:**

The mitochondrial genome of *S. variegata* was 587,383 bp in length and contained 52 genes, including 31 protein-coding genes (PCGs), 3 ribosomal RNAs (rRNAs), and 18 transfer RNAs (tRNAs). Analyses of relative synonymous codon usage (RSCU), repeat sequences, RNA editing events, and gene selective pressure (dN/dS) were conducted. Additionally, 7 chloroplast genes, including 4 PCGs, 2 tRNAs, and 1 rRNA, were identified within the mitochondrial genome. A preliminary phylogenetic analysis based on three mitochondrial genes suggested that *S. variegata* is most closely related to *S. suchowensis* and *S. purpurea*.

**Discussion:**

Overall, this study successfully assembled the mitochondrial genome of *S. variegata*, thereby enriching the mitochondrial genomic resources for willows and providing new insights into genetics, systematics, and mitochondrial evolution.

## 1 Introduction

*Salix variegata* Franch. is a perennial shrub in the genus *Salix* (Su et al., 2010), characterized by its typically oblong-oblanceolate or obovate-oblong leaves, rapid growth rate, robust reproductive capacity, and high biomass (Zhou et al., 2017). *S. variegata* is widely distributed in the northern part of Yunnan, Guizhou, Sichuan, Chongqing, the western part of Hubei, the southeastern part of Gansu, the southern part of Shaanxi, and Henan, among others in China. It’s renowned for aesthetic tree form and resilience to flooding, drought, and heavy metal stress (Chen et al., 2019; Luo et al., 2007; Sun et al., 2012). *S. variegata* serves as a common ornamental plant in gardens and is pivotal for soil stabilization and bank protection, underscoring its significant ornamental and ecological restoration values. The family Salicaceae, which includes important tree genera such as *Salix* and *Populus*, has been the focus of several phylogenetic and evolutionary studies. However, most of these studies have primarily concentrated on the chloroplast genome or nuclear markers, and the mitochondrial genome remains largely unexplored in this family. To date, only a limited number of complete mitochondrial genomes from Salicaceae species have been sequenced and assembled, hindering a comprehensive understanding of their evolutionary dynamics and molecular mechanisms (Qu et al., 2023). Recent advances in organelle genomics have begun to unravel the complex evolutionary dynamics within the family Salicaceae (Kim et al., 2025; Qu et al., 2023; Ren et al., 2021). They highlighted the necessity of incorporating mitochondrial genomic data for resolving phylogenetic relationships within Salicaceae. Although the chloroplast genomes of this species have been published (Xu et al., 2023), the mitochondrial genome (mt genome) has not yet been explored extensively, and there is a lack of a comprehensive study and analysis of the genomes of its cellular organelles.

Mitochondria serve as the site of oxidative metabolism in cells (Li et al., 2024) and play a crucial role in various biological processes, including cell growth, division, programmed cell death, and the synthesis of certain compounds, all of which are essential for plant growth (Chen et al., 2024). Plant mitochondrial (mt) genomes exhibit significant structural variation among species. The sequencing rate of plant mt genomes is considerably slower than that of chloroplast genomes, owing to the greater complexity of mt genomes (Liu D. et al., 2022). The size and structure of mt genomes vary greatly among different angiosperms. Usually, the size of mt genomes ranges from approximately 66 kb in the parasitic plant *Viscum scurruloideum* Barlow to 11.3 Mb in *Silene conica* L., with even larger genomes (up to 18.99 Mb) reported in some gymnosperms such as *Cathaya argyrophylla* Chun & Kuang (Huang K. et al., 2025; Skippington et al., 2015; Sloan et al., 2012), and the structure of plant mt genomes is varied, with a ring, linear, multi-branched, or several combinations of structures, such as *Platycladus orientalis* (L.) Franco, *Brasenia schreberi* J. F. Gmel., *Zea mays* L., and *Actinidia chinensis* Planch (Cardi et al., 2019; Fauron et al., 1995; Liu D. et al., 2022; Shan et al., 2024; Wang et al., 2019). The mt genome of *Lactuca sativa* L. is linear (Kozik et al., 2019), and the mt genomes of giant spruce *Picea sitchensis* (Bong.) Carrière and *Quercus acutissima* Carruth. are multibranched in structure (Jackman et al., 2020). The mt genomes in angiosperms contain abundant repetitive sequences that play a significant role in promoting homologous recombination, enhancing genomic diversity, driving evolutionary processes, and facilitating plant adaptation to environmental changes (Chevigny et al., 2020). According to the endosymbiotic theory, mitochondria originated from ancient endosymbiotic bacteria (Su et al., 2024). Plant mitochondria and chloroplasts usually follow the law of maternal inheritance, a genetic mechanism that eliminates the influence of the paternal lineage. That reduces the complexity of genetic studies and is more conducive to inferring intraspecific or interspecific phylogenetic relationships (Shan et al., 2024; Wang et al., 2019). Recent advances in sequencing technology have prompted a substantial increase in studies of plant mt genomes. These investigations primarily address species identification, interspecific phylogeny, intraspecific matrilineal evolution, population phylogeny, and genetic diversity (Kozik et al., 2019).

In this study, we first assembled and annotated the mitochondrial genome of *S. variegata*. We then analyzed its structural features and investigated its phylogenetic relationships. This work not only enriches our understanding of mitochondrial genomics in Salicaceae but also provides a theoretical basis for further studies on the cellular organelles of *S. variegata*.

## 2 Materials and methods

### 2.1 Plant material and mt genome sequencing

Fresh leaf samples of *S. variegata* were collected from the bank of the Jialing River in Beibei District, Chongqing, China (106.42092155°E, 29.84939019°N), immediately frozen in liquid nitrogen, and stored at −80 °C. Total DNA was extracted using the CTAB method (Arseneau et al., 2017). For second-generation (Illumina) sequencing, libraries were prepared following the standard protocol, including random fragmentation, end repair, A-tailing, adapter ligation, purification, and PCR amplification (Bolger et al., 2014; Emerman et al., 2017), and sequenced on the Illumina NovaSeq-6000 platform (Illumina, CA, USA). Raw reads were pre-processed with Trimmomatic to remove low-quality bases (Q < 19) and reads containing ambiguous “N” bases. For third-generation sequencing, libraries were prepared for the PacBio Sequel II platform (Pacific Biosciences, California, USA) using DNA damage repair, end repair, and adapter ligation without PCR amplification, and sequenced in Cyclic Consensus Sequencing (CCS) mode. A total of 22.85 Gb (77.07 ×) Illumina data and 21.17 Gb (~77 ×) CCS data were obtained.

### 2.2 Mt genome assembly and annotation

Raw Illumina and PacBio CCS (HiFi) reads were filtered using fastp (v0.23.4) (Chen et al., 2018) to remove adapters and low-quality bases. Mitochondrial reads were enriched by mapping clean reads to a reference set of five Salicaceae mt genomes (e.g., *Salix suchowensis* W. C. Cheng NC_029317.1, *Salix purpurea* L. NC_029693.1). Mapping was performed with BWA-MEM (v0.7.17) (Li, 2013) for Illumina reads and minimap2 (v2.26) (Li, 2018) with the map-hifi preset for PacBio HiFi reads. Reads that successfully mapped to the references (including their unmapped mates) were retained. *De novo* assembly was performed using GetOrganelle (v1.7.4.1) (Jin et al., 2020) for Illumina data and Flye (v2.9.1) with the --pacbio-hifi option for PacBio HiFi reads. The resulting assembly graph was visualized with Bandage (Wick et al., 2015), revealing a single circular-mapping molecule with no alternative branches or ambiguous structures (**Supplementary Figure 1**). The Flye-derived circular contig was polished using NextPolish (v1.4.0) (Hu et al., 2020) with two rounds of Illumina short reads. Inconsistencies between Flye and GetOrganelle contigs were manually inspected and resolved in IGV (v2.16.0) (Robinson et al., 2011), preferentially retaining long-read-supported bases. Initial genome annotation was performed using GeSeq and IPMGA (Tillich et al., 2017). tRNA genes were identified using tRNAscan-SE (Lowe and Eddy, 1997) with organellar settings. rRNA and protein-coding genes (PCGs) were annotated via BLASTn and BLASTp against the NCBI non-redundant database, focusing on closely related taxa. Manual curation was performed in Apollo to validate start/stop codons, intron boundaries, and rRNA termini (Lewis et al., 2002). The final mt genome map was drawn using OGDRAW (v1.3.1) (Alverson et al., 2010). For experimental validation, four primer pairs were designed targeting repeat regions and their flanking sequences using Primer-BLAST (accessed January 6, 2025; **Supplementary Table 1**). PCR was performed under standard conditions. PCR products were visualized on 1% agarose gels. Single bright bands were excised and Sanger-sequenced at Tsingke Bio-technology Co., Ltd.

### 2.3 Codon preference analysis

The protein-coding sequences (PCGs) of the mt genome were extracted using PhyloSuite software (version 1.2.2; Zhang et al., 2020). PhyloSuite was chosen for this initial step due to its robust batch-processing capabilities for extracting sequences from mitochondrial genome annotations. Although PhyloSuite integrates comprehensive pipelines for phylogenetic analysis, our study focused specifically on codon usage bias rather than phylogeny reconstruction. Therefore, we utilized only the sequence extraction module of PhyloSuite, which provides a reliable foundation for downstream codon usage analysis. Following established criteria (sequence length > 300 bp, start codon AUG, and termination codons UAG, UAA, or UGA). Exclude interference from start and stop codons that arise solely from RNA editing events. A final set of 31 protein-coding sequences was selected for subsequent analysis. Detailed analysis of codon usage patterns, including the calculation of Relative Synonymous Codon Usage (RSCU), was performed using MEGA 11.0 software (Kumar et al., 2016), which offers specialized functions for codon-based evolutionary analysis. Data visualization and mapping were performed using RStudio. This study primarily focused on calculating and interpreting RSCU values to characterize codon usage bias in the mt genome. The effective number of codons (ENC) values and GC content at the third synonymous codon position (GC3s) were calculated using CodonW (v1.4.4) (Grote et al., 2005).

### 2.4 Mt genome repeats sequence analysis

In this study, three types of repetitive sequence analysis were performed: simple sequence repeats, tandem repeats, and dispersed repeats. Simple sequence repeats were analyzed using MISA (https://webblast.ipk-gatersleben.de/misa/, accessed on September 26, 2024) (Beier et al., 2017). The minimum number of occurrences of each type of nucleotide repeats was: single nucleotide repeats at least ten times, dinucleotide repeats at least five times, trinucleotide repeats at least four times, and tetranucleotide, pentanucleotide, and hexanucleotide repeats at least three times. Scattered repeat sequences were performed using REPuter (https://bibiserv.cebitec.uni-bielefeld.de/reputer, accessed September 26, 2024) (Kurtz et al., 2001). Tandem repeat sequences were visualized using the Tandem Repeats Finder (TRF) tool (https://tandem.bu.edu/trf/trf.html, accessed on September 26, 2024) (Benson, 1999), followed by the Advanced Circos software package (Zhang et al., 2013).

### 2.5 Mitochondrial plastid homologous DNA sequence analysis (MTPTs)

The chloroplast genome sequence of *S. variegata* (GenBank accession number: NC_057289.1; Chen, 2019) was downloaded from the NCBI database. Homologous regions between the mitochondrial and chloroplast genomes were identified using BLASTn (version 2.12.0+; Chen et al., 2015) with an E-value cutoff of 1e-10, visualized via TBtools (Basic Circos module; Chen et al., 2020), and each fragment was counted once regardless of the inverted repeat duplication. Functional potential was assessed via BLASTx against plastid protein-coding sequences: an intact open reading frame without internal stop codons or frameshifts, and the presence of conserved domains (CDD) indicated potential functionality, whereas frameshifts or internal stop codons indicated pseudogenization. Putative promoters were predicted using PlantProm DB (release 2.0; core promoter score ≥ 0.8) in the 500 bp upstream region (or entire shorter intergenic region). Secondary structures of putatively non-coding or untranslated regions were predicted with RNAfold (ViennaRNA package, version 2.4.14) using the minimum free energy algorithm (default parameters). Relative transfer timing was estimated via synonymous substitution rates (dS): alignments with MAFFT (version 7.475) and dS calculation with Yn00 (PAML version 4.9), with higher dS interpreted as older transfers (noting the lack of a reliable molecular clock). All functional, pseudogenization, promoter, structural, and timing analyses are computational predictions and lack experimental validation.

### 2.6 RNA editing site prediction

The RNA editing events in the mt genome of *S. variegata* were predicted using the Plant Mitochondrial Gene RNA Editing Prediction Tool on the Sangsin Cloud Platform (http://cloud.genepioneer.com:9919/#/login). This prediction process required extracting all mitochondrial protein-coding sequences from the assembled mitochondrial genes, after which these sequences were uploaded to the BioSignal Cloud Platform for analysis. We considered predictions with probability values greater than 0.9 to be reliable.

### 2.7 Analysis of nucleotide substitution rate

The 25 PCGs shared across five Salicaceae species were extracted from the mt genome annotations of *S. variegata*, *S. suchowensis*, *S. purpurea*, *Populus alba* L., and *Populus tremula* L. using PhyloSuite v1.2.2 (Zhang et al., 2020). Multiple sequence alignments for each gene were performed using MAFFT v7.505 (Katoh and Standley, 2013) with default parameters. Alignments were manually inspected and trimmed to ensure codon integrity. Pairwise nonsynonymous substitution rates (dN), synonymous substitution rates (dS), and their ratio (ω = dN/dS) were calculated for each orthologous gene pair using the YN00 module implemented in PAML v4.10.7 (Yang, 2007). Statistical significance of selection (ω < 1, ω = 1, or ω > 1) was assessed using Fisher’s Exact Test as implemented in the YN00 algorithm, with p < 0.05 considered indicative of significant deviation from neutrality. To further investigate potential episodic positive selection along specific lineages, we performed branch-site model analysis using the Codeml program in the PAML package. The branch leading to the genus *Salix* (i.e., the common ancestor of *S. variegata*, *S. suchowensis*, and *S. purpurea*) was designated as the foreground branch, with the genus *Populus* (*P. alba* and *P. tremula*) set as background branches. We compared Model A (allowing positive selection on the foreground branch) against the corresponding null model (fixing ω = 1 on the foreground branch) using likelihood ratio tests (LRTs). Statistical significance was evaluated against a chi-square distribution with one degree of freedom, with p < 0.05 considered evidence for positive selection. All resulting dN/dS values were visualized using TBtools v2.008 (Chen et al., 2023).

### 2.8 Collinearity analysis

To investigate the structural conservation and rearrangement patterns of the *S. variegata* mt genome relative to other angiosperms, we performed pairwise collinearity analyses with four representative species. Three phylogenetic distance levels were considered: congeneric (*Salix suchowensis* NC_029317.1, *S. purpurea* NC_029693.1), confamilial within the same order (Malpighiales) but different families – Euphorbiaceae represented by *Manihot esculenta* Crantz (NC_045136.1), and a more distantly related outgroup from a different order – *Arabidopsis thaliana* (L.) Heynh. (Brassicaceae, JF729202.1). These selections allow us to quantify how collinearity retention decays with increasing phylogenetic divergence, rather than merely confirming known relationships. Whole−genome alignments were performed using BLASTn v2.13.0 (Camacho et al., 2009) with an E−value threshold of 1e−10 and a minimum alignment length of 100 bp to ensure detection of conserved syntenic blocks. The resulting BLAST outputs were processed using the MCscanX pipeline (Wang et al., 2012) implemented in TBtools v2.008 (Chen et al., 2023) to identify and visualize collinear blocks between *S. variegata* and each of the four reference genomes. Genomic regions showing conserved collinearity were defined as blocks containing at least four homologous gene pairs with consistent order and orientation. Rearrangement events were inferred when collinear blocks appeared in different orders or orientations between species. The distribution of conserved collinear gene fragments shared across all five species was identified by intersecting pairwise collinearity results. All collinear plots were visualized using the Multiple Synteny Plot module in TBtools, with collinear blocks colored to highlight conservation patterns across different phylogenetic distances.

### 2.9 Phylogenetic analysis

To assess the phylogenetic position of *S. variegata*, conserved PCGs were retrieved from the mt genomes of 30 species closely related to *S. variegata* (selected based on phylogenetic affinity) plus one outgroup, *Amborella trichopoda* Baill., all downloaded from NCBI. Homologous sequences were extracted and identified using PhyloSuite (version 1.2.2; Zhang et al., 2013). Only three PCGs - *rps12*, *rps3*, and *rps4* - were included in this analysis. This limited selection was necessary because these were the only genes that could be reliably aligned without frameshifts, internal stop codons, or excessive missing data across all 31 compared taxa. The remaining 38 conserved angiosperm mitochondrial PCGs were excluded due to alignment ambiguity, incomplete sequence availability, or pseudogenization in one or more of the study species. Homologous sequences of the three selected genes were aligned using MAFFT (version 7.471; Katoh and Standley, 2013), and the alignments were concatenated. Ambiguous alignment regions were trimmed with Gblocks (Talavera and Castresana, 2007) using a minimum block length of 10. Maximum-likelihood (ML) phylogenetic analysis was performed with IQ-TREE (Nguyen et al., 2015) using 16 threads and 1,000 bootstrap replicates. The best-fit substitution model was selected by the Bayesian Information Criterion (BIC), which identified “TVM+F+G4” as the optimal model. The resulting tree was visualized and refined using MEGA 11 and ITOL (https://itol.embl.de/; Letunic and Bork, 2019). We emphasize that this phylogenetic inference is based on an extremely limited gene set (n=3) and should therefore be considered preliminary and hypothesis-generating, not a definitive species-level resolution.

## 3 Results

### 3.1 Structural characterization of the mt genome of *S. variegata*

**Figure 1:**
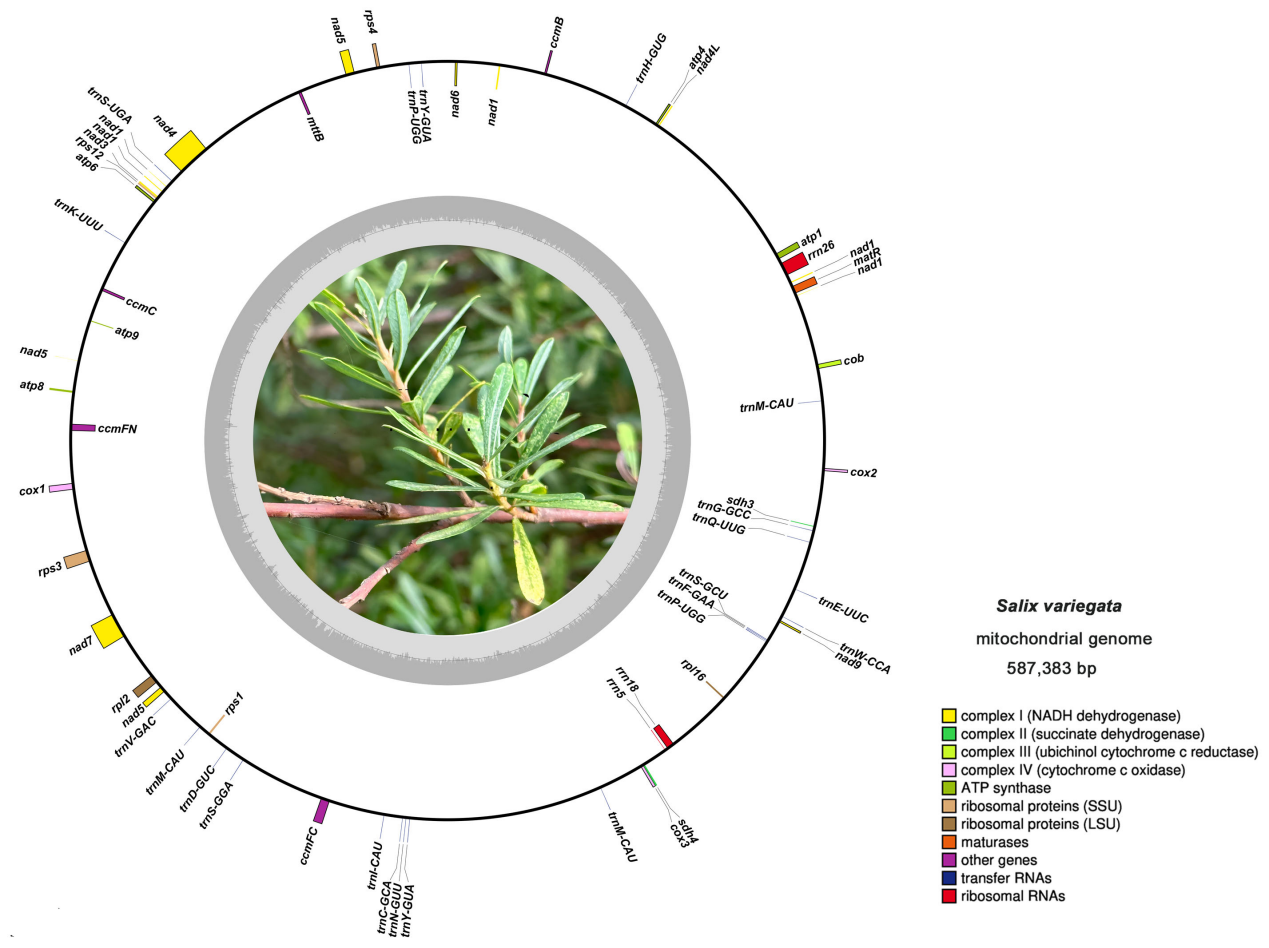
The circular mt genome map of *S. variegata*. Genes of different functional groups are annotated using different colors.

The mt genome of *S. variegata* has a circular-mapping molecule with a total length of 587,383 bp and a GC content of 44.91% (**Figure 1**). Visualization of the assembly graph with Bandage revealed a simple, unbranched circular contig, indicating the absence of alternative conformations or unresolved repetitive regions that often complicate plant mitochondrial assemblies. To further validate the junctions spanning repetitive sequences, PCR amplification using four specific primer pairs (**Supplementary Table 1**) yielded single products of the expected sizes, and Sanger sequencing of these amplicons confirmed the precise nucleotide sequences across the repeat regions. This combined computational and experimental approach confirms the reliability of the assembled mt genome structure. PCR results showed bands of the expected size (**Supplementary Figure 1**; **Supplementary Figure 2**).

Annotation of the *S. variegata* mt genome identified a total of fifty-two unique genes, comprising thirty-one protein-coding genes (PCGs), three rRNA genes, and eighteen tRNA genes (**Supplementary Table 2**). Following the conceptual framework for plant mt genome diversity outlined by Mower et al. (2012), these PCGs can be categorized into core genes involved in oxidative phosphorylation and variable genes (primarily ribosomal proteins and succinate dehydrogenase) that exhibit lineage-specific retention or loss across angiosperms. The core gene set in *S. variegata* includes twenty-four genes that are highly conserved across plant mt genomes: five ATP synthase genes (*atp1*, *atp4*, *atp6*, *atp8*, *atp9*), four cytochrome c biogenesis genes (*ccmB*, *ccmC*, *ccmFC*, *ccmFN*), nine NADH dehydrogenase genes (*nad1*, *nad2*, *nad3*, *nad4*, *nad4L*, *nad5*, *nad6*, *nad7*, *nad9)*, three cytochrome c oxidase genes (*cox1*, *cox2*, *cox3*), one cytochrome c reductase gene (*cob*), one maturase gene (*matR*), and one transporter gene (*mttB*). The variable gene set comprises seven genes: five ribosomal protein genes (*rpl2*, *rpl16*, *rps1*, *rps3*, *rps4*), the *rps12* gene (which functions in the ribosome small subunit but shows variable genomic location across species), and one succinate dehydrogenase gene (*sdh4*). Comparative analysis with recently published Salicaceae mt genomes reveals that the gene content of *S. variegata* is largely canonical for the family. The mt genome of *Salix psammophila* C. Wang & C. Y. Yang contains thirty-three PCGs (twenty-four core, nine variables), while *S. suchowensis* encodes thirty-two PCGs (Qiao et al., 2024; Ye et al., 2017). Notably, similar to other Salicaceae species, *S. variegata* lacks the *rps14* gene—a characteristic hypothesized to be associated with the evolution of stress tolerance within this family. The variable nature of ribosomal protein genes observed here—with *S. variegata* retaining *rpl2*, *rpl16*, *rps1*, *rps3*, *rps4*, and *rps12*—is consistent with broader patterns of ribosomal gene loss and retention documented across angiosperms. The genome also encodes three ribosomal RNAs (*rrn5*, *rrn18*, *rrn26*) and eighteen tRNA genes capable of recognizing codons for all amino acids (**Supplementary Table 2**). Intron analysis identified eight introns distributed across various genes, a number comparable to other Salicaceae mt genomes (e.g., seventeen introns in *S. suchowensis*, with differences reflecting intron gain/loss dynamics typical of plant mitochondrial evolution).

### 3.2 Codon preference analysis

**Figure 2:**
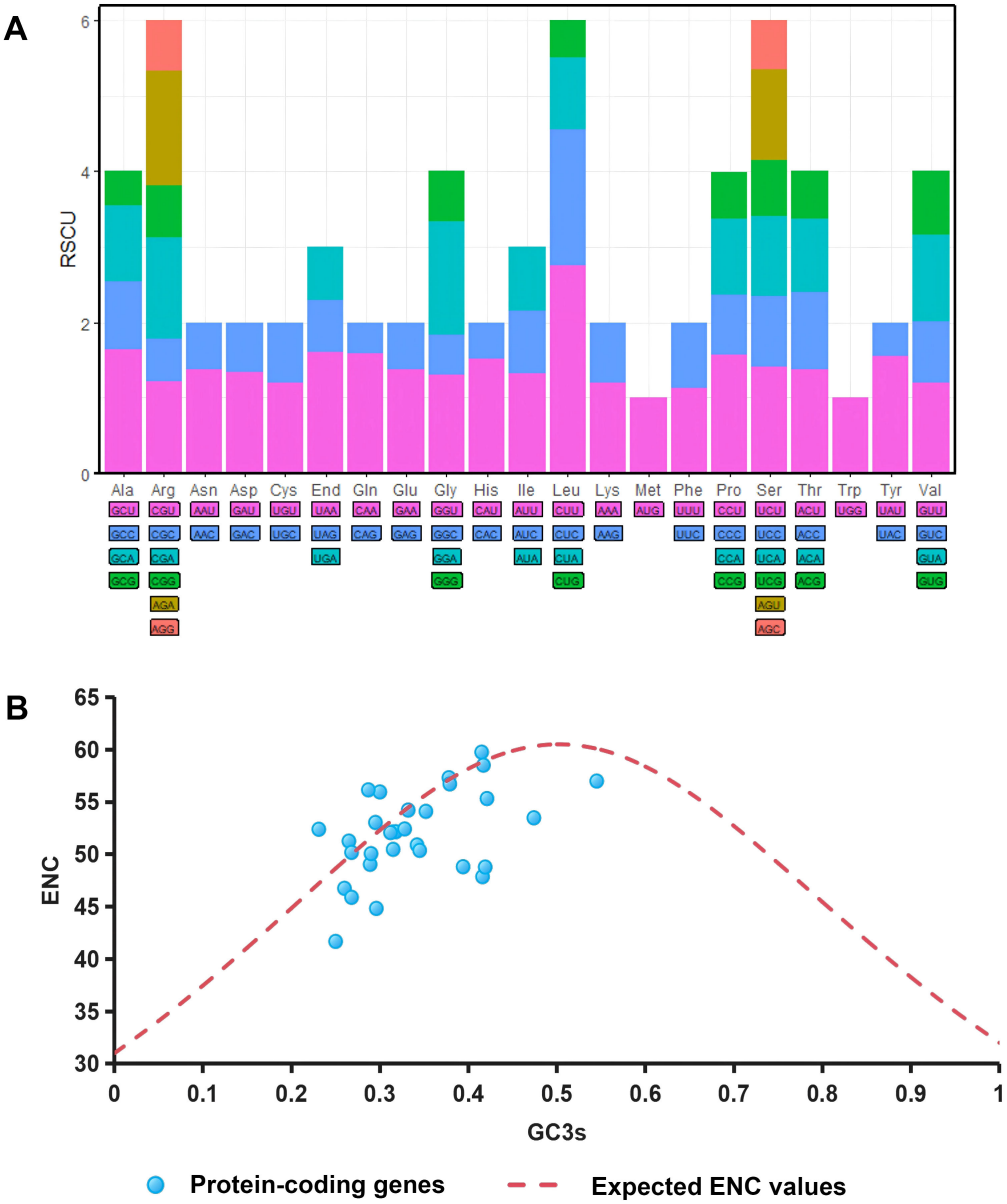
Codon preference of *S. variegata* mt genome. **(A)** Depict the distribution of RSCU values in the mt genome. The *x-axis* shows the codon group, and the relative synonymous codon usage (RSCU) is the ratio of the observed frequency of codon usage to the expected usage of that codon. **(B)** The ENC-GC3 plot of *S. variegata* mitochondrial genes. Each dot represents a single protein-coding gene. The red curve indicates the expected ENC value under mutation pressure alone.

In this study, we analyzed the codon usage of 31 PCGs of the mt genome that satisfy the criteria, and the codon usage of each amino acid is shown in **Supplementary Table 3**. The results indicated that the protein-coding sequences exhibited significant codon usage bias. Among these, alanine demonstrated a pronounced preference for GCU, with the highest relative synonymous codon usage (RSCU) value of 1.64. This was followed by the termination codon UAA, which had an RSCU value of 1.6. Glutamine also showed a significant preference for CAA, with an RSCU value of 1.58. The codons with an unbiased RSCU value of 1 were tryptophan codon UGG, proline codon CCA, and methionine codon AUG (**Figure 2A**). Analysis of 64 codons in the mt genome of *S. variegata* revealed that there were 31 codons with a higher usage frequency (RSCU ≥ 1), among which 90.3% (28/31) ended with A/U bases. This indicates that the codons used in the mitochondrial DNA of *S. variegata* tend to end with A/U bases. This characteristic is not unique to *S. variegata*; it has also been observed in the chloroplast genome of the *Salix matsudana* Koidz. in the Salicaceae family (Zou et al., 2026). Overall, most amino acids in the protein-coding sequences of the *S. variegata* mt genome show biased codon usage. ENC-GC3s analysis can further help understand the underlying drivers behind codon usage (Li et al., 2023). In the organelle of *S. variegata*, most of the PCGs were distributed on or near the standard curve (**Figure 2B**). The genes *rpl16*, *rps1*, *atp8*, and *sdh4* were farthest from the standard curve. Analyzing these codon preferences helps elucidate the underlying patterns, offering valuable references for predicting gene expression levels and studying evolutionary trends.

### 3.3 *S. variegata* mt genome repeat sequence analysis

**Figure 3:**
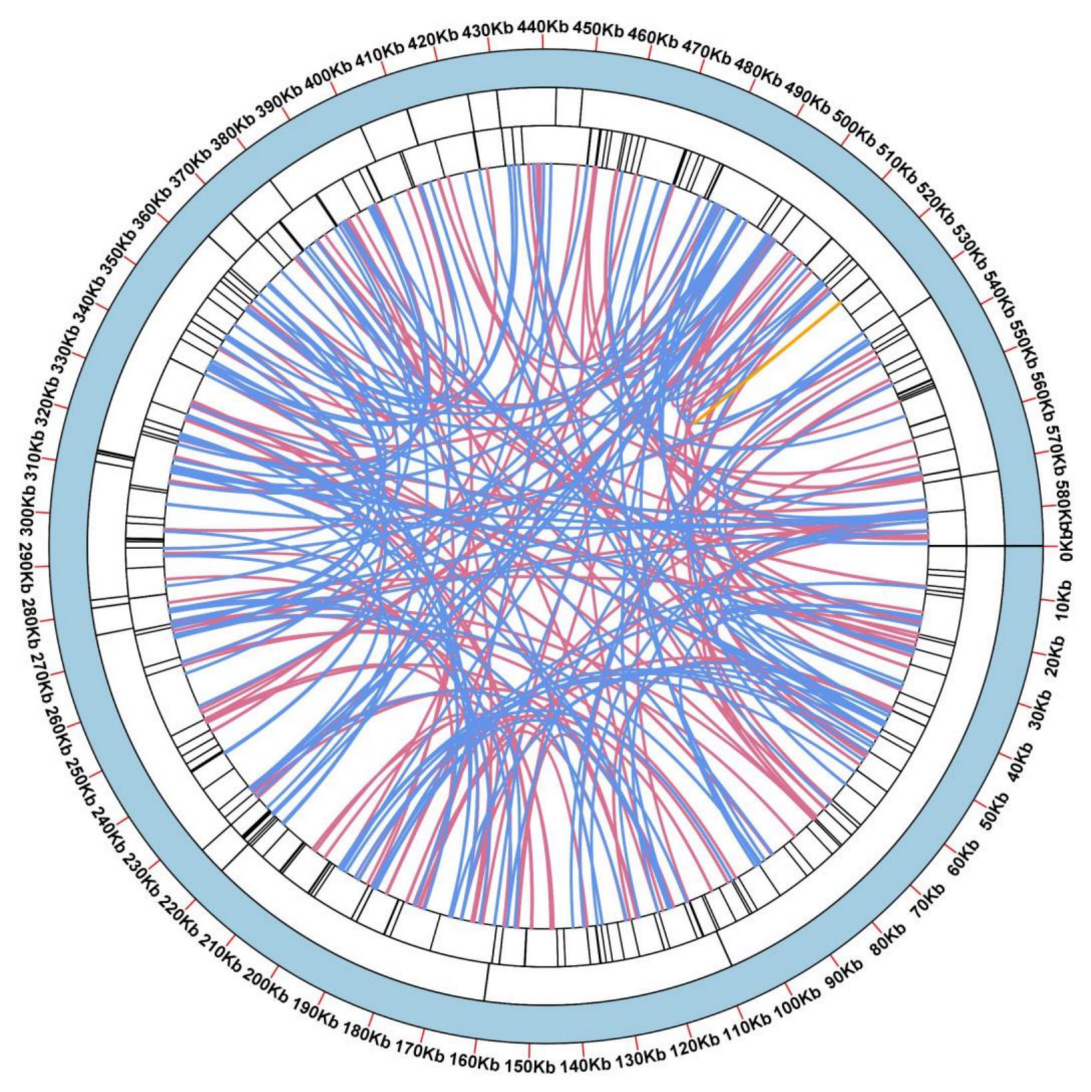
Analysis of repeat sequences in the *S. variegata* mt genome. It depicts the spatial distribution of repeat sequences inside the *S. variegata* mt genome. In the innermost circle, pairs of dispersed repeats are connected by colored lines, the red for palindromic repeats, blue for forward repeats, and orange for inverted repeats. The black line segments in the middle and the red line on the outermost circles denote the locations and lengths of tandem repeats and SSRs, respectively.

In this study, we analyzed the mt genome of *S. variegata* for three kinds of repetitive sequences: simple sequence repeats, tandem repeats, and scattered repeats (**Figure 3**). We identified 189 SSRs, with more single-nucleotide repeats present, 69 in total, accounting for 36.51%, followed by 61 tetranucleotide repeats, 28 dinucleotide and trinucleotide repeats, 21, respectively, and relatively few pentanucleotide repeats, 9, and one hexanucleotide repeat (**Supplementary Table 4**). We also detected 22 tandem repeats (**Supplementary Table 5**), and setting the size criterion of the minimum repeat sequence to 100 bp, 276 dispersed repeats were identified (**Supplementary Table 6**), which included 146 forward repeats, 129 palindromic repeats, and one inverted repeat was also detected. The total number of SSRs in *S. variegata* (189) is significantly higher than in *Salix babylonica* L. (142) and *S. suchowensis* (151), primarily driven by the expansion of mononucleotide repeats, reflecting lineage-specific mutation rates (Han et al., 2022). Scattered repeats play a critical role in mediating genomic recombination. We identified 276 scattered repeats in *S. variegata*, predominantly forward (146) and palindromic (129) repeats. Notably, a pair of 248 bp direct repeats identified in the *atp9-nad3* intergenic region may serve as potential recombination hotspots, mediating genomic structural variation through homologous recombination. Similar recombination hotspots have been confirmed as a conserved mechanism of structural evolution in various plant mitogenomes (Gualberto and Newton, 2017; Woloszynska, 2010). The tandem repeat sequences are unstable in organisms and frequently undergo mutations. They are involved in the regulatory activities of the genome and are closely related to genome recombination and rearrangement. The repeat sequences of different species of the genus *Salix* vary greatly. Only three species (*Salix Cardiophylla* Trautv. & C.A.Mey., *Salix Paraflabellaris* S. D. Zhao, and *S. suchowensis*) have repeat sequences longer than 1,000 bp (Han et al., 2022; Kim et al., 2025). The functional significance of short and long repeats appears to be distinct. Short repeats, such as SSRs, primarily serve as molecular markers, and their length polymorphisms may influence the expression of adjacent genes. In contrast, long scattered repeats (>100 bp) are more frequently involved in genomic rearrangements and structural variation. In this study, 62.3% of the scattered repeats were located in intergenic regions. This distribution pattern may represent a buffering mechanism developed during genome evolution to mitigate the potentially deleterious effects of repeat insertion on coding sequences, thereby maintaining genome stability while preserving structural plasticity (Rajendrakumar et al., 2007). The repeat content identified in *S. variegata* aligns with the broader patterns reported by Kim et al. (2025) across 11 *Salix* species, who found that mono- and tetranucleotide SSRs consistently dominate *Salix* mitogenomes. However, the total SSR count in *S. variegata* (189) exceeds the average reported for the genus (162 ± 15), primarily due to expansion of mononucleotide repeats. This variation falls within the intrageneric diversity documented by Kim et al., supporting their conclusion that repeat content variation is a major driver of mt genome diversity in *Salix*.

### 3.4 Mitochondrial plastid homologous DNA sequence analysis (MTPTs)

**Figure 4:**
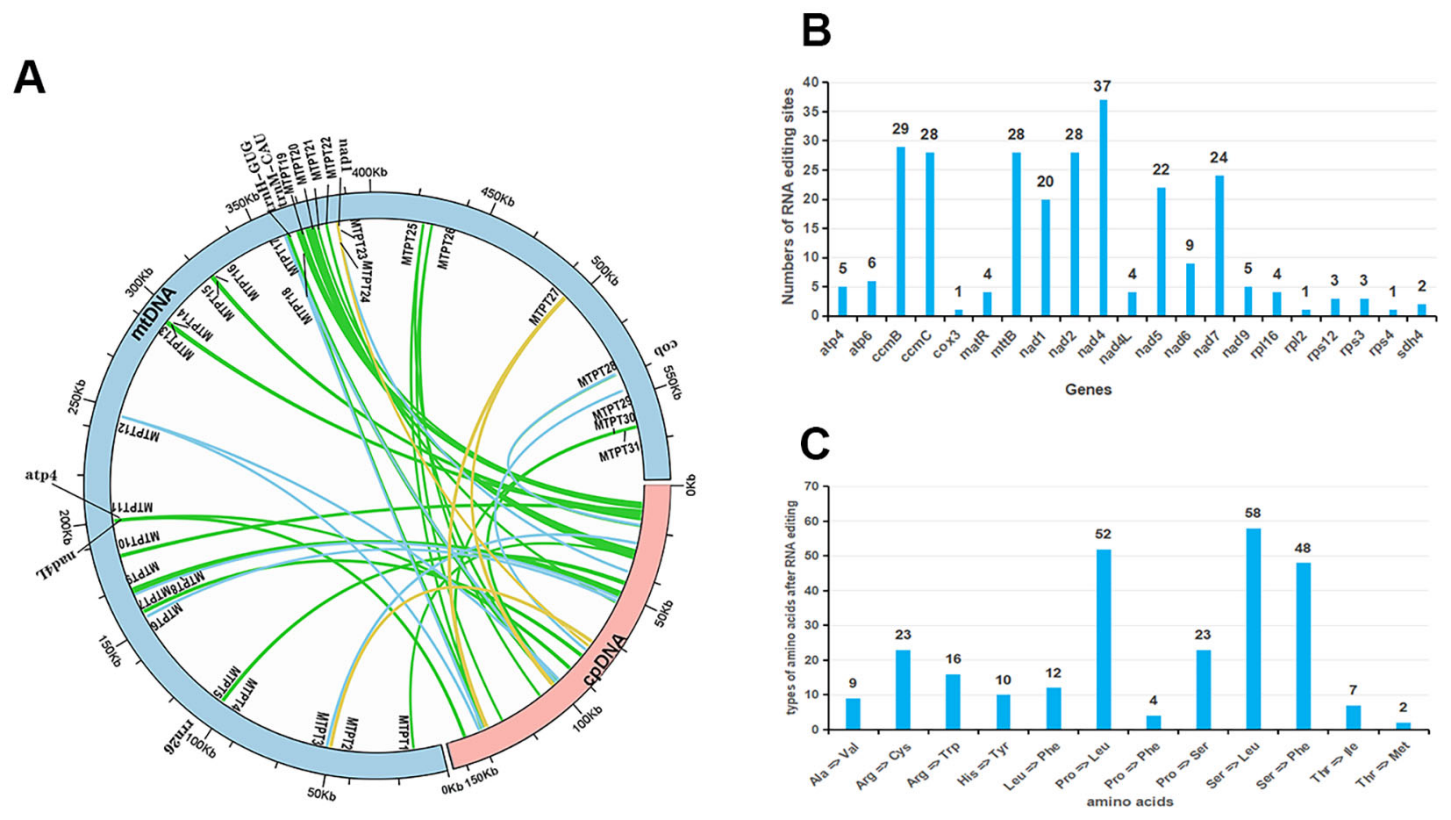
**(A)** Homologous sequence analysis of *S. variegata* mt genome and chloroplast genome. Visualization of BLAST results of *S. variegata* chloroplast genome and mt genome. The dark green line segments indicate sequence similarity greater than 90%, the blue line segments indicate sequence similarity greater than 80%, and the yellow color indicates sequence similarity greater than 70%. MTPTs represent mitochondrial plastid DNA. **(B)** Number of RNA editing sites in the mt genome. **(C)** Effect of RNA-editing on amino acid translation.

Using BLASTn homology search, we identified 31 homologous sequences between the chloroplast and mt genomes of *S. variegata*, with a total length of 22,103 bp. These sequences represent putative mitochondrial plastid DNAs (MTPTs; plastid-derived DNA fragments that have been transferred to the mt genome) and account for approximately 3.76 % of the mt genome and 14.19 % of the chloroplast genome (**Figure 4A**; **Supplementary Table 7**). Based on these sequence data alone, this finding suggests that the chloroplast genome may serve as a potential source of DNA sequences for the mt genome of *S. variegata*. The longest fragment, MTPT20, was 3,542 bp in length. Further computational characterization revealed that plastid-derived fragments have been integrated into seven canonical mitochondrial genes through predicted intracellular gene transfer (IGT) events. These include four protein-coding genes (*nad4L*, *atp4*, *nad1*, *cob*), two tRNA genes (*trnH-GUG*, *trnM-CAU*), and one rRNA gene (*rrn26*). As previously documented in angiosperm mt genomes, plastid-derived DNA can contribute to existing mitochondrial genes via gene conversion or insertion into intronic regions, sometimes becoming functional components of chimeric genes (Cai et al., 2026). The presence of these MTPT fragments within canonical mitochondrial genes may reflect the dynamic nature of inter-organellar DNA transfer and ongoing intracellular gene migration (Park et al., 2020; Zhu et al., 2025). Among these sequences, some are incomplete—truncated, disrupted by internal stop codons, or present only as partial fragments. Computationally, this tentatively suggests that they may represent non-functional residues of once-migrated chloroplast DNA that have undergone degradation and pseudogenization over evolutionary time (Watanabe et al., 1994; Woloszynska et al., 2004; Szandar et al., 2022; Zhao et al., 2023). The number of MTPTs identified in *S. variegata* (seven) falls within the frequency range (4–12 MTPTs per species) reported by Kim et al. (2025) across 11 *Salix* species. Our predictions that *trnH-GUG* and *trnM-CAU* may retain functionality (based on intact ORF and conserved domain criteria), while protein-coding MTPTs are pseudogenized, are consistent with Kim et al.’s observation that tRNA genes are preferentially retained as functional transfers in *Salix* mitogenomes. This pattern provides preliminary support for the hypothesis that functional constraints differ substantially between tRNA and protein-coding genes following inter-organellar transfer. Finally, based on synonymous substitution rate (dS) estimates (which are a rough computational approximation), the relative timing of transfer events in *S. variegata* was preliminarily estimated to be approximately 6-8 mya. This tentatively falls within the diversification window of the genus and is broadly consistent with Kim et al.’s suggestion that MTPT accumulation in *Salix* has been an ongoing process throughout genus evolution. We emphasize that all functional, pseudogenization, and timing conclusions are derived from in silico predictions and have not been experimentally validated (e.g., by transcript detection, RT-PCR, or functional assays).

### 3.5 RNA editing site prediction

In this computational study, a total of 266 RNA editing sites were predicted in 31 mitochondrial PCGs of *S. variegata* using a probability cutoff (**Supplementary Table 8**). All predicted editing sites were C-to-U (C-to-T in DNA) transitions, and their distribution across each protein-coding gene is shown in **Figure 4B**. Among these genes, *nad4* exhibited the highest number of predicted editing sites (37), followed by *ccmB* (29), whereas *cox3*, *rpl2*, and *rps4* each possessed only one predicted site. In this predictive analysis, RNA editing events that lead to premature formation of stop codons were found, for example, in the *atp4* gene, the CAA codon was predicted to be edited to UAA. In addition, RNA editing is also critical for creating start codons, such as the *nad1* and *nad7*, where the ACG codon was predicted to be edited to AUG. These RNA editing events will affect subsequent amino acid translation. Under our analysis parameters, all 266 predicted editing sites resulted in predicted non-synonymous amino acid changes, while no synonymous C-to-U editing events were identified in this computational study. The most frequent predicted conversion was from serine (Ser) to leucine (Leu), occurring 58 times (21.8% of all predicted editing events). Within the context of our computational predictions, the prevalence of non-synonymous editing events suggests a potentially important role for RNA editing in protein translation in the *S. variegata* mt genome (**Figure 4C**).

### 3.6 Analysis of differences in the mt genome gene composition of five species of Salicaceae

**Figure 5:**
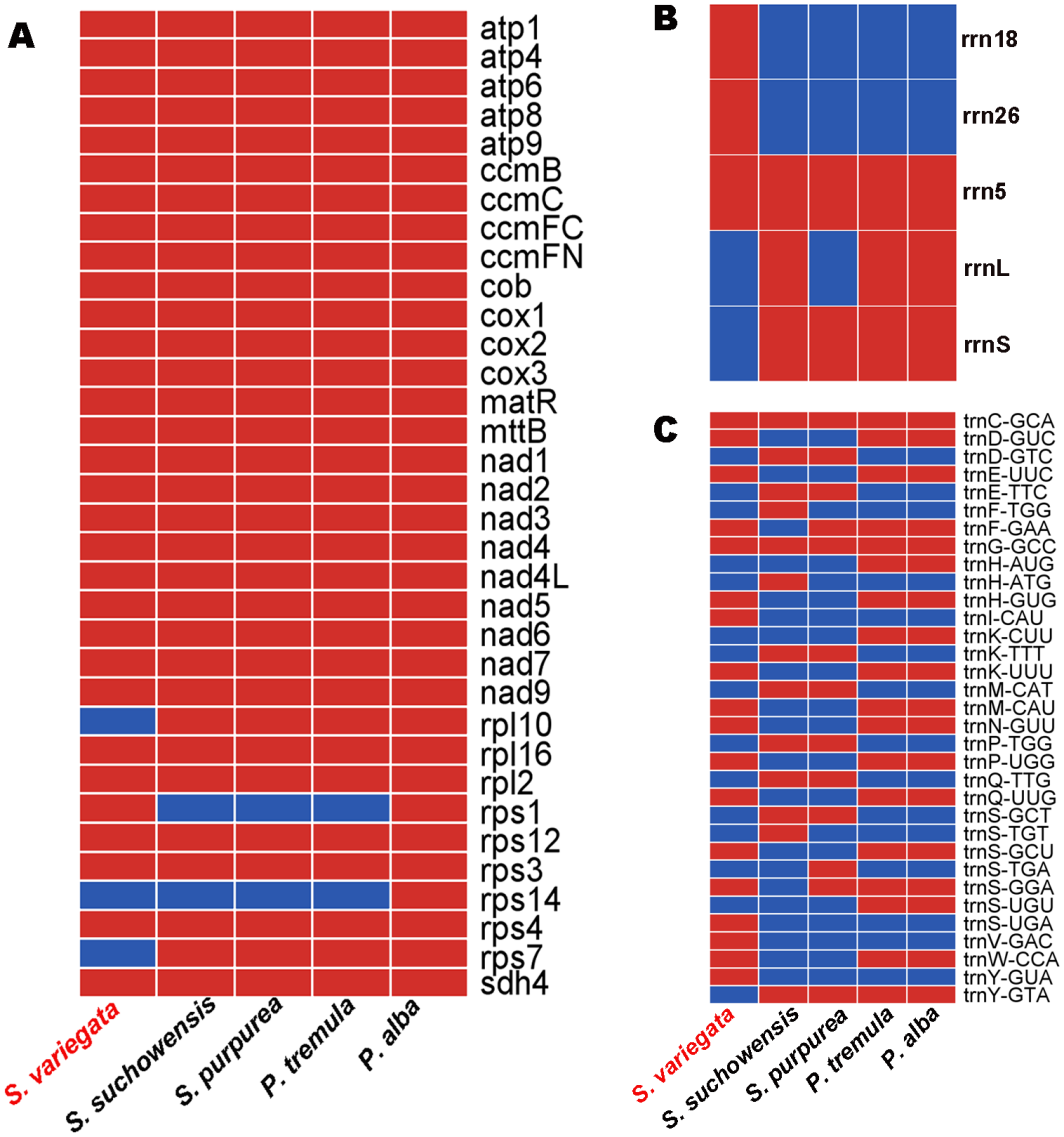
**(A)** Composition of PCGs; **(B)** rRNAs; and **(C)** tRNAs in the mt genomes of five species of Salicaceae.

Compared the gene composition of mt genomes across five Salicaceae species and determined that *P. alba* contained the highest number of PCGs, with 34, followed by *S. purpurea*, *S. suchowensis*, and *P. tremula*, each with 32 PCGs. *S. variegata* possessed the fewest PCGs, with only 31 (**Figure 5A**). The rRNA statistics for the mt genomes of the five species revealed that all four species had three rRNAs, except for *S. purpurea*, which had two rRNAs (**Figure 5B**). Finally, the tRNA annotation statistics for the mt genomes showed that *S. variegata*, *P. alba*, and *P. tremula* all had eighteen tRNAs, and the same number of tRNAs were present in *S. purpurea* and *S. suchowensis*, both with thirteen (**Figure 5C**).

### 3.7 Variation in substitution rates of PCGs in the mt genomes of five species of Salicaceae

**Figure 6:**
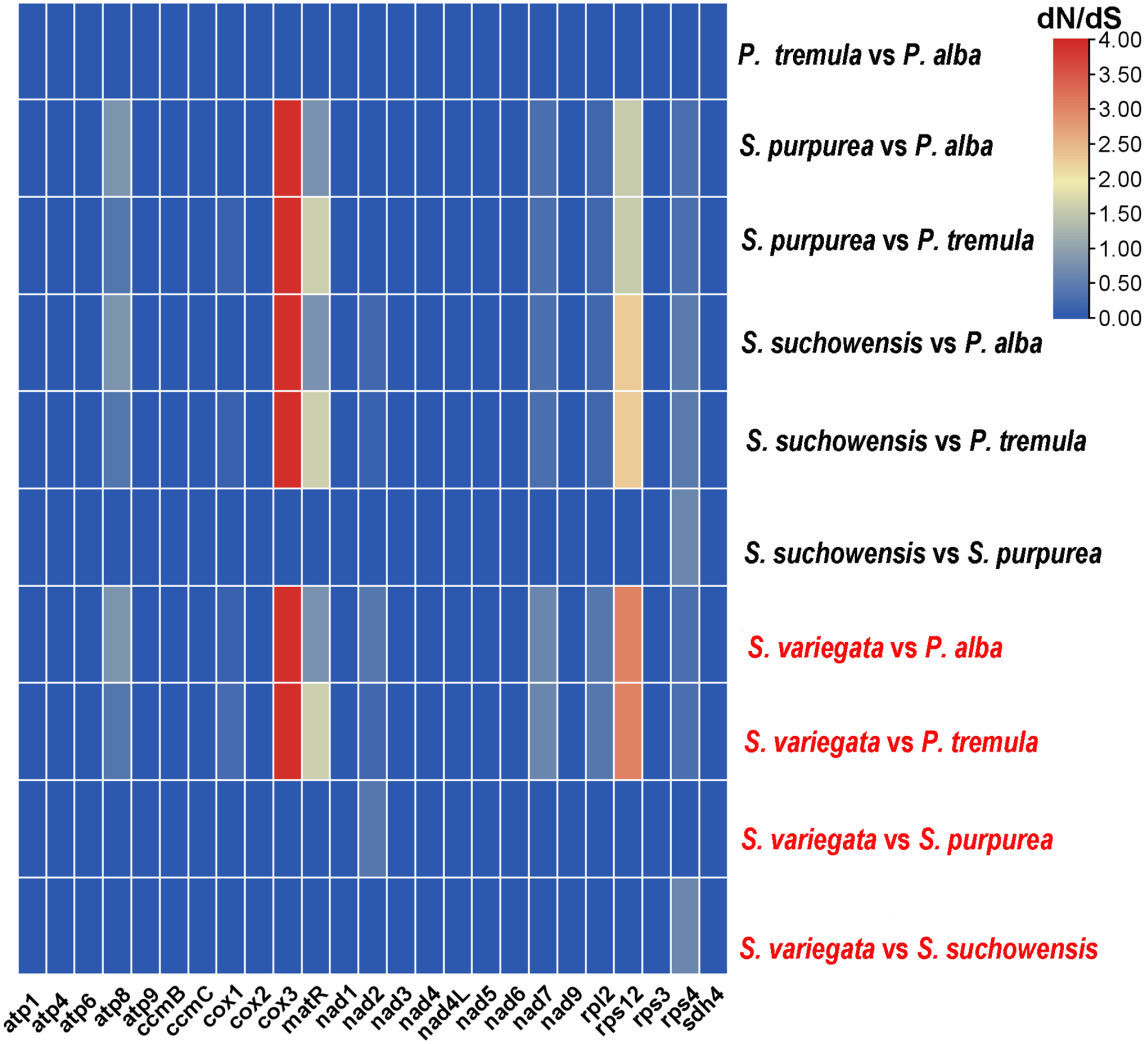
Heat map of dN/dS values of 25 shared genes in the mt genomes of five species of Salicaceae plants.

To investigate the evolutionary constraints acting on the mitochondrial genomes of Salicaceae species, the twenty-five shared protein-coding genes (PCGs) were extracted from the mt genomes of *S. variegata* and four other Salicaceae species (*S. suchowensis*, *S. purpurea*, *P. alba*, and *P. tremula*). Pairwise dN (nonsynonymous substitution rate), dS (synonymous substitution rate), and their ratio (ω = dN/dS) were calculated for each gene to infer the mode and strength of selection. The results showed that the ω values for the vast majority of genes were significantly less than 1 (p < 0.05, Fisher ‘s Exact Test) across all pairwise comparisons, indicating that they have been subjected to strong purifying selection acting to conserve their functions. Notably, three genes—*cox3* (cytochrome c oxidase subunit 3), *matR* (maturase R), and *rps12* (ribosomal protein S12)—exhibited ω values exceeding 1 in several species pairwise comparisons (e.g., *S. variegata* vs. *S. suchowensis*) (**Supplementary Table 9**). However, statistical evaluation using Fisher’s Exact Test revealed that these ω > 1 values were not significantly different from neutrality (p > 0.05). Therefore, while these genes show a trend consistent with relaxed purifying selection or very weak positive selection, the null hypothesis of neutral evolution (ω = 1) cannot be rejected based on pairwise comparisons alone. To further explore whether specific amino acid sites along a particular lineage have experienced positive selection, we employed the branch-site model (Model A) in the PAML (Codeml) package. We set the branch leading to the genus *Salix* as the foreground branch. A Likelihood Ratio Test (LRT) comparing Model A (which allows positive selection) against the null model (ω fixed to 1) was not significant for *cox3*, *matR*, or *rps12* (p > 0.05), confirming the absence of robust statistical support for positive selection in these genes (**Figure 6**; **Supplementary Table 9**).

### 3.8 Collinearity analysis

**Figure 7:**
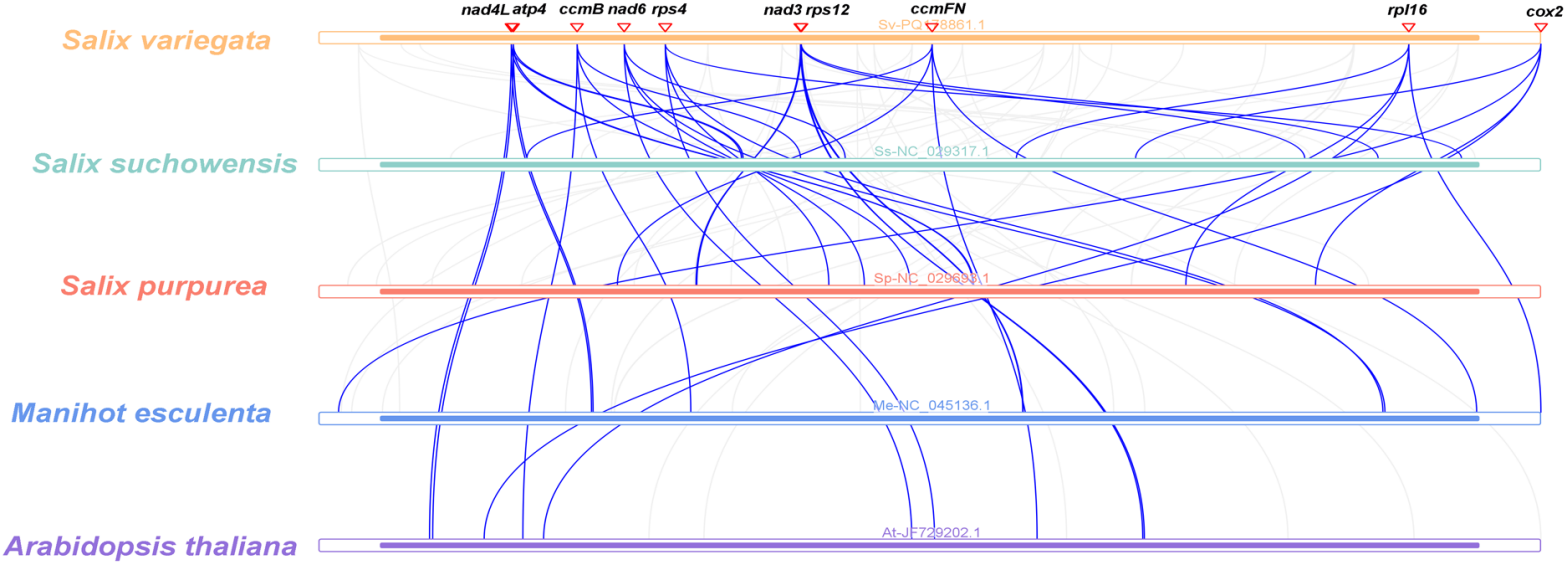
Analysis of the covariance of the *S. variegata* mt genome with the four species; the dark blue highlighted line segments are covariance segments shared by the five species.

We performed collinearity analysis of the *S. variegata* mt genome against those of *S. suchowensis*, *S. purpurea*, *M. esculenta*, and *A. thaliana* (**Figure 7**; **Supplementary Table 10**). Results showed more conserved collinearity regions between *S. variegata* and its congeners *S. suchowensis* and *S. purpurea*, indicating their close phylogenetic relationship. Conversely, fewer collinearity regions existed between *S. variegata* and the Euphorbiaceae and Brassicaceae families, suggesting greater phylogenetic distance. In addition, the size of the mt genomes of these species varied significantly, with the mt genomes of *M. esculenta* and *S. suchowensis* exceeding 600 Kb, compared with the smaller mt genome of *A. thaliana*, which was about 350 Kb. A total of ten gene fragments were detected in the *S. variegata* mt genome, all of which represent conserved collinear regions shared with the other four plant species. There were multiple co-linear regions between the mt genomes of *S. variegata* and those of closely related species (**Supplementary Figure 3**). The results showed that these conserved collinear regions were arranged in different orders in the mt genomes of different species, which suggests that the mt genomes of *S. variegata* may have been subjected to a more extensive genomic rearrangement with closely related species.

### 3.9 Phylogenetic analysis

**Figure 8:**
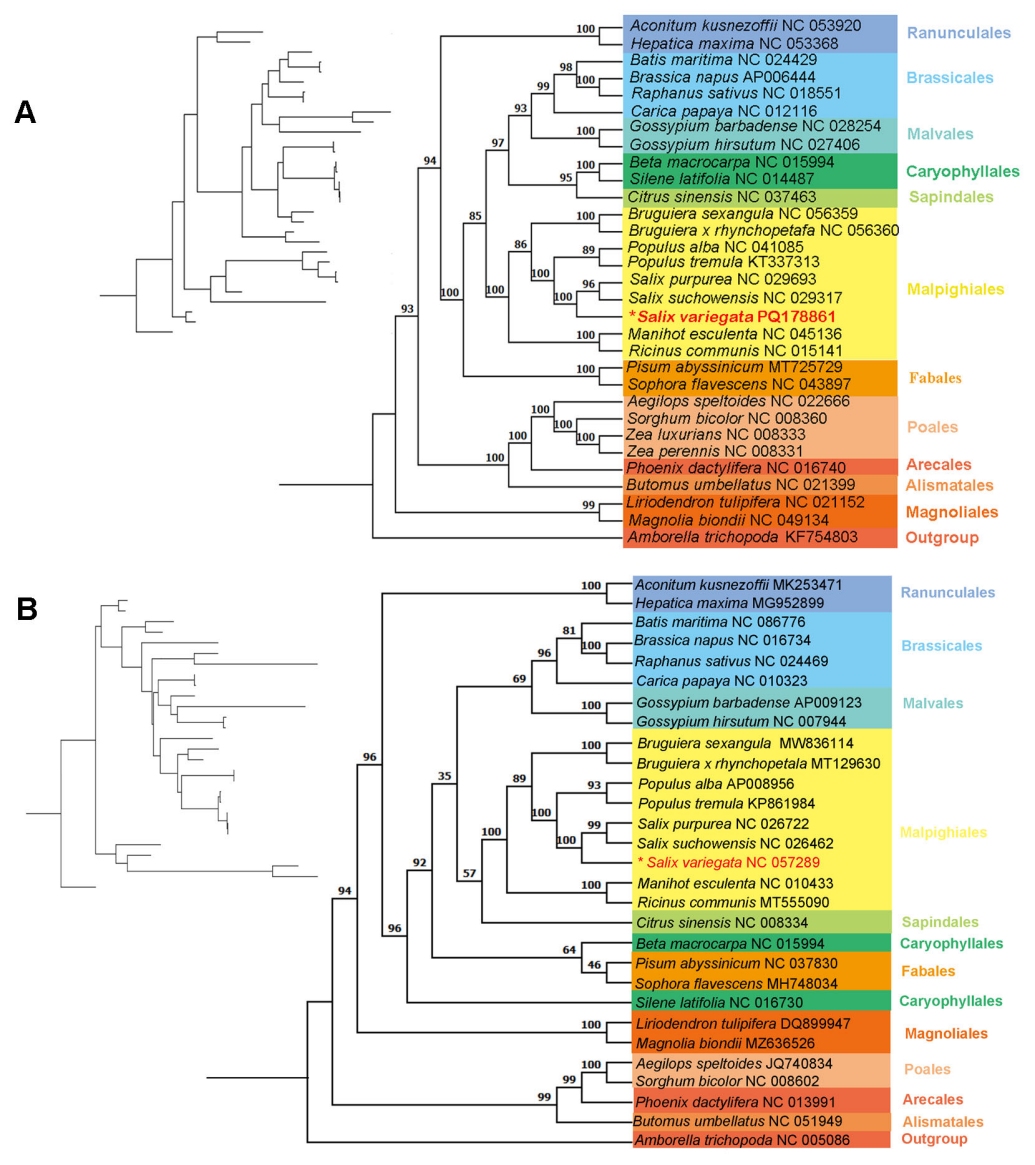
Phylogenetic tree. **(A)** Phylogenetic tree of 31 angiosperms based on conserved mitochondrial genomic protein-coding sequences. *A. trichopoda* was selected as an outgroup, and the numbers at each node are bootstrap values. **(B)** Phylogenetic tree of 29 angiosperms based on conserved chloroplast genomic protein-coding sequences. *A. trichopoda* was selected as an outgroup, and the numbers at each node are bootstrap values.

Mitochondria and chloroplasts are crucial organelles in plant cells, each possessing semi-autonomous genomes independent of the nuclear genome. Using only three mitochondrial PCGs (*rps12*, *rps3*, and *rps4*) - due to alignment and availability constraints as described in Methods - we constructed a preliminary phylogenetic tree of 31 angiosperm species (**Figure 8A**; **Supplementary Table 11**). We emphasize that this analysis is based on an extremely limited gene set and should therefore be interpreted as hypothesis-generating rather than conclusive. Within this preliminary mitochondrial tree, 25 out of 28 nodes exhibited bootstrap support values >90%, of which 16 nodes showed 100% bootstrap support. However, high bootstrap support on a small number of genes does not guarantee accurate phylogenetic resolution, and these values are reported here for completeness without overinterpretation. A parallel tree was constructed using chloroplast genome sequences from the same species (**Figure 8B**; **Supplementary Table 12**). When compared, the mitochondrial and chloroplast trees showed some topological similarities, but this comparison is limited by the narrow gene set used for the mitochondrial inference. Specifically, in the mitochondrial tree, the Malpighiales branch appeared closely related to the Sapindales and Caryophyllales branches, whereas in the chloroplast tree, Malpighiales appeared more closely related to Sapindales alone. In the mitochondrial tree, *S. variegata* showed relatively close genetic distance to *S. suchowensis* and *S. purpurea*, followed by *P. alba* and *P. tremula*. All four species belong to the Salicaceae family, which is consistent with the current Angiosperm Phylogeny Group (APG) classification and previous molecular and taxonomic studies (Ren et al., 2021). Nevertheless, this consistency does not validate the three-gene tree as robust; a larger matrix of conserved mitochondrial PCGs (e.g., 20–41 genes) would be required to confidently resolve the phylogenetic position of *S. variegata*.

## 4 Discussion

### 4.1 Mitochondrial genome characteristics of *S. variegata*

Mitochondria are vital organelles in eukaryotic cells, providing energy for various physiological and metabolic activities. The plant mt genome is more complex than that of animals, with extensive variation in genome size, abundant repetitive sequences, and highly conserved coding sequences (Cheng Y. et al., 2021). In this study, the *S. variegata* mt genome was assembled as a circular-mapping molecule, and relative synonymous codon usage (RSCU) was analyzed to examine its codon usage pattern. RSCU is defined as the ratio of the observed frequency of a codon to its expected frequency under equal usage (Shields et al., 1988). An RSCU value >1 indicates that the codon is used more frequently than expected (a high-frequency codon); RSCU = 1 indicates unbiased usage (i.e., codons are selected randomly or equally during RNA transcription); RSCU <1 indicates that the codon is used less frequently (Wang et al., 2018). Codon usage bias is considered an important phenomenon in plant evolution, and analysis of codon preference in *S. variegata* may help to understand the molecular mechanisms underlying its adaptive evolution to the environment (Barbhuiya et al., 2019; Novembre, 2002). In our analysis, the codon GCU showed the highest RSCU value (1.64). The majority of preferred codons ended in A or U, suggesting a significant bias in codon selection in *S. variegata*. This characteristic is not unique to this species; for example, codon preference analysis of the mitochondrial genome of *S. psammophila* also revealed a relatively high preference for the codons UAA, ALA, and GCU (Qiao et al., 2024). Similar studies on codon usage in mt genomes have been reported for other angiosperms, such as *Houttuynia cordata* Thunb. and *Primula mallophylla* Balf. F (Huang Y. et al., 2025; Li et al., 2025). We also observed that some mitochondrial-encoded genes in willows show both higher expression levels and stronger codon usage preference, which could be tentatively related to energy allocation demands. However, we caution that this is a *post-hoc* inference; functional studies are needed to test any causal link.

Repetitive sequences are widely present in plant mitochondrial genomes (Gualberto et al., 2014) and are usually essential for intermolecular recombination. Repetitive sequence-mediated homologous recombination is prevalent in plant mt genomes (Cheng L. et al., 2021; Odahara et al., 2021; Woloszynska, 2010). The size of repetitive sequences is closely related to recombination frequency (Varré et al., 2019), with short repeats generally associated with lower recombination frequency than long repeats. For example, higher recombination frequency mediated by long repetitive sequences has been reported in *Nymphaea colorata* Peter and *Scutellaria tsinyunensis* C. Y. Wu & S. Chow, whereas short repetitive sequences exhibited lower recombination frequency in those species (Dong et al., 2018; Li et al., 2021). In the mt genome of *S. variegata*, most repetitive sequences were short repeats, which typically exhibit lower recombination frequencies. While this feature may contribute to genome stability, the claim of “high genetic stability” remains speculative without quantitative support. The long-dispersed repeats identified in the mt genome of *S. variegata*, particularly the paired forward repeats, may serve as potential recombination hotspots. Comparative analyses with other willow species showed a tentative positive correlation between repeat sequence abundance and phylogenetic distance. Given the lack of quantitative validation, we refrain from concluding a causal role for repeat-driven recombination in species differentiation. Future work with expanded sampling and statistical testing is needed. In plants, RNA editing (primarily cytosine-to-uracil conversion) is common in mitochondrial and chloroplast genomes and can influence gene expression (Mader et al., 2020). The number of RNA editing sites reported in mt genomes varies among species: approximately 491 in *Oryza sativa* L (Clifton et al., 2004), about 486 in *Phaseolus vulgaris* L (Bi et al., 2020), and around 330 in *S. suchowensis* (Ye et al., 2017). Similarly, we computationally predicted 266 RNA editing sites in the mt genome of *S. variegata*, all of which were predicted to be C-to-U transitions. It should be emphasized that these predictions have not been validated experimentally (e.g., by transcriptome or RT-PCR). As such, the predicted RNA editing sites provide a computational basis for hypothesizing potential effects on gene expression of the alternative genetic code, but such hypotheses require empirical confirmation. Analysis of dN/dS values can increase the understanding of the mt genome evolution in Salicaceae. The results showed that most of the genes in these five species of Salicaceae had dN/dS values less than 1, which may imply that these genes are conserved (Liu H. et al., 2022; Ran et al., 2018). However, we also found that the dN/dS ratios for *cox3*, *matR*, and *rps12* were greater than 1. Further statistical testing using branch−site models indicated that ω > 1 for these three genes was not statistically significant (P > 0.05). Therefore, there is no evidence supporting positive selection acting on these genes during the evolution of the five Salicaceae species. The elevated dN/dS values more likely reflect relaxed selective constraints or genetic drift rather than genuine adaptive evolution. These three genes are involved in the biosynthesis of cytochrome C oxidase, maturation enzymes, and ribosomal proteins, respectively. The evolutionary analysis of mitochondrial PCGs revealed that purifying selection is the predominant force maintaining the functional integrity of essential genes involved in oxidative phosphorylation and translation. This is consistent with the general view that organellar genomes experience strong functional constraints (Wolfe et al., 1987). The origin of terrestrial plants is thought to be associated with many horizontal transfer events from bacteria to aquatic organisms (Shan et al., 2023). In angiosperms, significant genetic interactions occur between the two organellar genomes, mainly in the form of chloroplast DNA fragments being transferred to and integrated into the mitochondrial genome. For example, the plastid-originated *rpl32* gene in the subfamily Thalictroideae was transferred to the nuclear genome (Park et al., 2015), and the *atpI* gene found in the mt genome of *Aeginetia indica* L. is thought to have originated in the chloroplast genome of another angiosperm. It has also been shown that genes transferred from plastids may not function in mitochondria and become pseudogenes during evolution (Choi and Park, 2021). Positioning our findings within the comparative framework established by Kim et al. (2025) reveals complementary insights into *Salix* mt genome evolution. The breadth-oriented analysis by Kim et al. (2025) across 11 species documented interspecific variation in MTPT content and established evolutionary patterns at the genus level. Our depth-oriented computational analysis of *S. variegata* complements this by providing an in-silico characterization of individual MTPTs, including predicted promoter regions, predicted secondary structures, and computationally assessed pseudogenization (see Methods 2.5). Together, these two approaches tentatively suggest that *Salix* mitogenomes are shaped by ongoing inter-organellar transfer, but that most protein-coding MTPTs are predicted to be rapidly pseudogenized, whereas tRNA genes show computational evidence for a higher probability of functional integration. This differential retention pattern could reflect stronger purifying selection on tRNA genes due to their essential role in mitochondrial translation and the difficulty of replacing them with nuclear-encoded alternatives, although experimental validation is required to confirm functionality. In this study, we analyzed sequence transfer between the chloroplast and mt genomes of *S. variegata*. Consistent with previous reports, we observed multiple sequence transfers between the two genomes. A total of seven chloroplast-derived genes were identified computationally, which suggests that the chloroplast genome may be a major source of transferred sequences for the mt genome. If functionally integrated and expressed, such transfers could potentially contribute to the genetic diversity of the *S. variegata* mt genome. However, we emphasize that all conclusions regarding MTPT functionality, retention, pseudogenization, and impact on genetic diversity are based on computational predictions (sequence homology, ORF integrity, promoter prediction, and substitution rates) and have not been experimentally validated. Therefore, they should be considered hypothesis-generating rather than definitive.

### 4.2 Phylogenetic status analysis of *S. variegata*

Salicaceae is a diverse family of plants. Compared to other genera in this family, such as *Idesia* and *Itoa*, the genera *Salix* and *Populus* contain more species (Huang et al., 2017). The genus *Salix* has about 450–580 species, and the genus *Populus* contains more than 100 species across six major sections (Turanga, Leuce, Leucoides, Aigeiros, Tacamahaca, and Abaso; Fang et al., 1999). These plants are widely distributed throughout the world except Oceania and Antarctica and constitute one of the major groups of trees and shrubs in the Northern Hemisphere (Argus et al., 2010). They are commonly used for horticultural ornamentation, protective forests, timber, papermaking, and a wide range of wood products (Karp and Shield, 2008). The large number of species in Salicaceae makes their classification and evolutionary relationships still challenging. Therefore, robust phylogenetic studies are needed. For most higher plants, chloroplast and mt genomes are maternally inherited, which reduces the complexity of genetic studies and facilitates interspecific phylogenetic analysis. However, studies on Salicaceae phylogeny based on mt genomes remain scarce. In this study, we assembled and annotated the mt genome of *S. variegata*. Using three PCGs (*rps12*, *rps3*, and *rps4*) from the mt genomes of 31 angiosperm species, we constructed a preliminary phylogenetic tree (as described in Methods). We emphasize that this tree is based on an extremely limited gene set and should not be interpreted as a robust species-level phylogeny. The tree tentatively placed *S. variegata* close to *S. suchowensis* and *S. purpurea*, and this observation is broadly consistent with the collinearity analysis (which showed conserved syntenic blocks between *S. variegata* and these two congeners). However, the consistency does not validate the three-gene tree, and a larger set of mitochondrial PCGs would be required for confident inference. Previous phylogenetic studies in Salicaceae have used chloroplast genome sequences or fragments thereof (e.g., *atpF-H*, *rbcL*, *trnL-F*, *matK*, *psbI-psbK*) (Li et al., 2015; Yun et al., 2015). In our chloroplast genome-based tree, *S. variegata* also appeared closely related to *S. suchowensis* and *S. purpurea*, which is in agreement with prior reports (Kim et al., 2025; Lauron-Moreau et al., 2014). Additionally, molecular marker studies using *ITS*, *matK*, and *rbcL* have evaluated phylogenetic relationships among 107 *Salix* species, including *S. purpurea* (Lauron-Moreau et al., 2014). Morphologically, *S. variegata*, *S. suchowensis*, and *S. purpurea* are all shrubs with alternate simple leaves, catkin inflorescences, and cottony seeds (Fang et al., 1999). Collectively, these independent lines of evidence (collinearity, chloroplast phylogeny, molecular markers, and morphology) are consistent with a relatively close relationship among the three species. Nevertheless, our mitochondrial phylogenetic contribution is limited by the use of only three genes. Therefore, we refrain from claiming that the mt genome alone reveals the phylogenetic position of *S. variegata* or lays a foundation for further genetic studies. Instead, we recommend that future studies employ a broader matrix of conserved mitochondrial PCGs (e.g., the 41-gene angiosperm core set) to achieve a reliable phylogenetic resolution within Salicaceae.

## 5 Conclusions

In this study, we successfully assembled and annotated the complete mt genome of *S. variegata*. The genome is a circular-mapping molecule of 587,383 bp, containing 52 genes with a GC content of 44.91%. Codon usage analysis revealed an obvious bias in codon selection. Most of the identified repetitive sequences were short repeats. While this observation is consistent with a relatively stable genome architecture, it does not directly demonstrate “high genetic stability”, which would require quantitative support (e.g., mutation rate analysis). Analysis of dN/dS values across five Salicaceae species showed that most genes have values <1, suggesting predominant purifying selection and functional conservation. Although the dN/dS ratios for *cox3*, *matR*, and *rps12* exceeded 1, branch-site model tests were not statistically significant (P > 0.05); thus, no evidence of positive selection was detected. The elevated ω values more likely reflect relaxed selective constraints or genetic drift. In addition, a preliminary phylogenetic tree was constructed using only three PCGs (*rps12*, *rps3*, and *rps4*) from the mt genomes of 31 angiosperm species. We acknowledge that this limited gene sampling restricts the robustness of the phylogenetic inference. Nevertheless, this preliminary analysis provides some reference information for exploring the phylogenetic position of *S. variegata* within the Salicaceae family. For future studies aiming to resolve the phylogenetic position of *S. variegata* within Salicaceae, we recommend using a substantially larger set of conserved mitochondrial PCGs (e.g., 20–41 genes) in combination with other genomic markers.

## Data Availability

The datasets presented in this study can be found in online repositories. The names of the repository/repositories and accession number(s) can be found below: https://www.ncbi.nlm.nih.gov/genbank/, PQ178861.1 https://ngdc.cncb.ac.cn/gsa/, PRJCA026796.
