# Supplementary material for: Complete mitochondrial genome of Salix variegata Franch.: assembly, characterization, and comparative analysis
Source: Front Plant Sci. 2026 Jun 19;17:1804453. doi: 10.3389/fpls.2026.1804453 (PMC13328496; doi:10.3389/fpls.2026.1804453)
Supplement: Supplementary file 1 [file Table1.docx]

**Supplementary Figures**


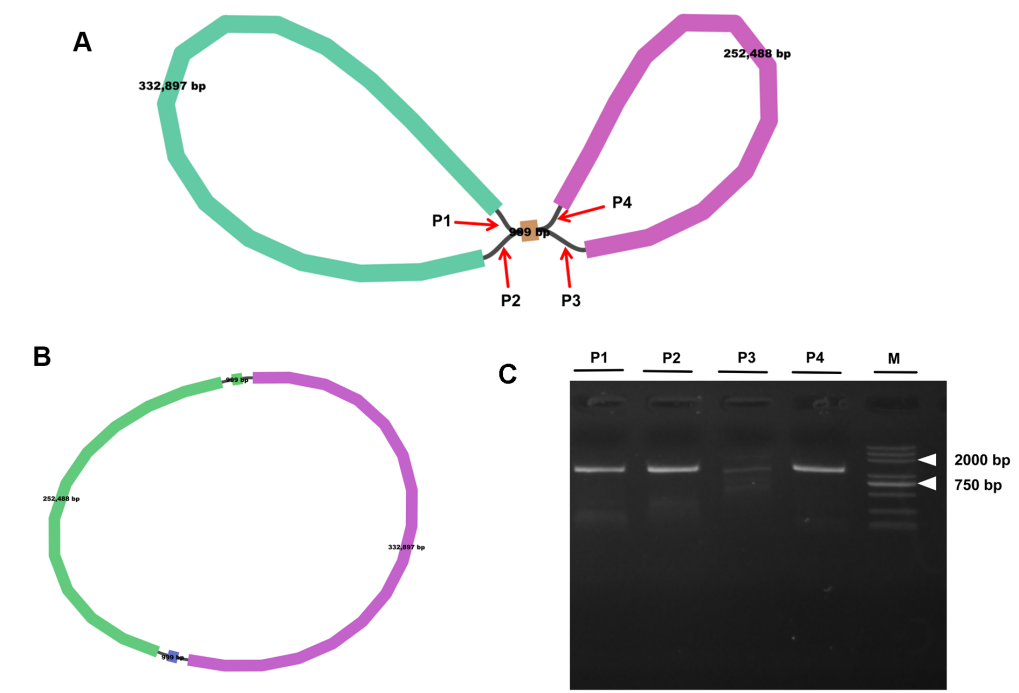


Figure S1. The assembly of S. variegata mt genome. (A) The mitogenome consists of three contigs with different lengths, and they connected to each other. (B) The potential master circle structure. (C) The electropherogram of 2 paths.

、


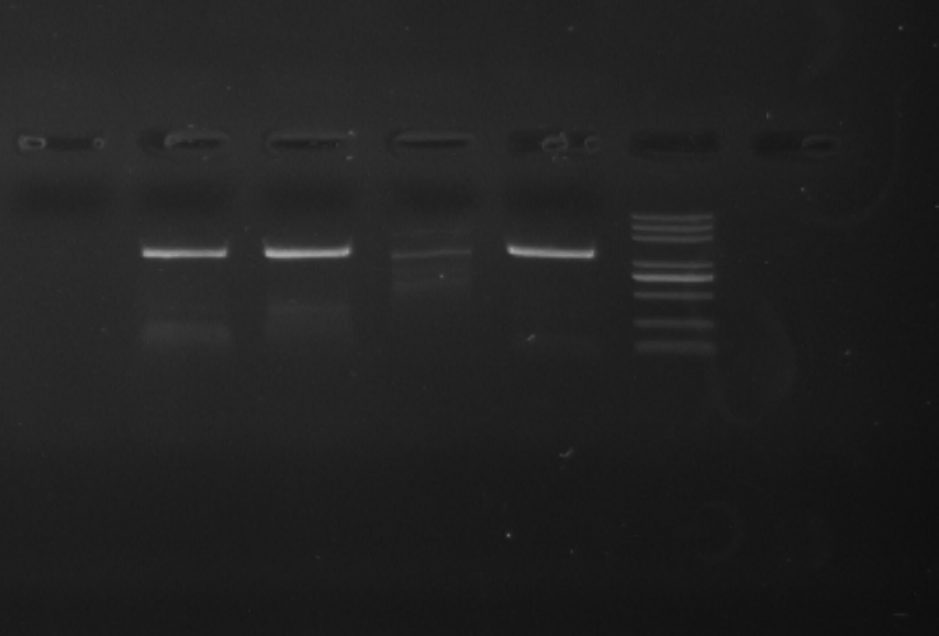


Figure S2. Raw amplification gel map.


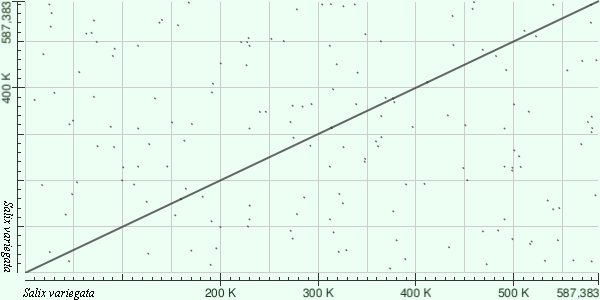


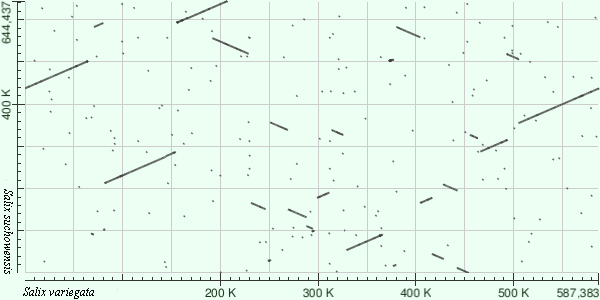


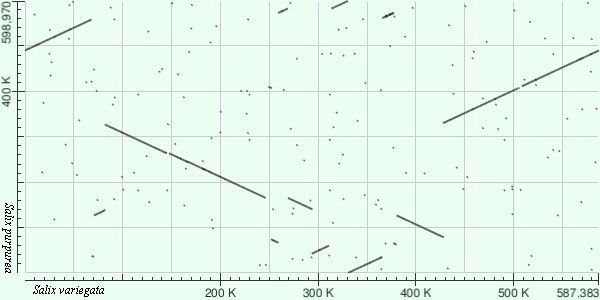


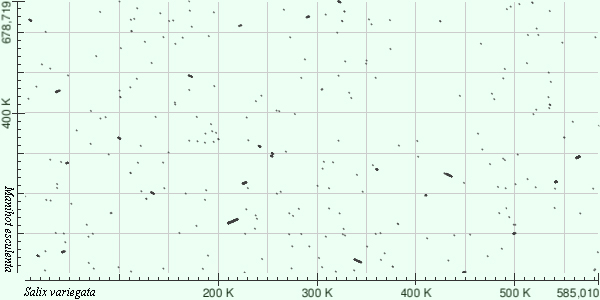


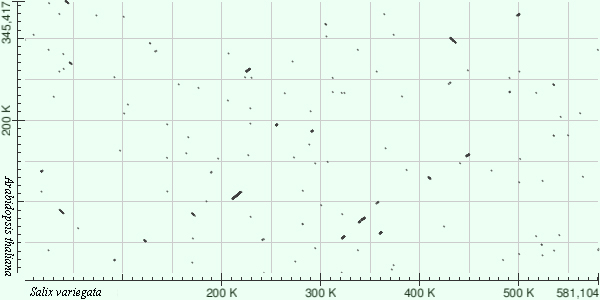


**Figure S3.**The self-dotplot of the mitogenome of *Salix variegata* and the dotplot of *Salix variegata* and its related species.
